# Some Notes on the Laboratory Side of Eugenics

**Published:** 1913-01

**Authors:** B. G. R. Williams

**Affiliations:** Paris, Ill., Author of “Laboratory Methods, with Special Reference to the Needs of the General Practitioner”


### Some Notes on the Laboratory Side of Eugenics.

BY B. G. R. WILLIAMS, M. D., PARIS, ILL., AUTHOR OF "LABORATORY METHODS, WITH SPECIAL REFERENCE TO THE NEEDS OF THE GENERAL PRACTITIONER."

Whether the practitioner is ready or no, the time is at hand when individual selfishness must yield to general weal. Whatever views he has entertained in the past concerning the needs of his patients and his private practice must be shelved as we approach the subject of eugenics, the application of which demands the use of precise diagnostic aids.

Pray how can the practitioner qualify in this work if he ignore all laboratory methods? He can no longer diagnosticate gonorrhea merely by an inquiry concerning the morning drop or a brief glance at the urine for clap-threads, but must search diligently for the gonococcus and prove that this is absent before he can certify that the man is free from the disease and the marriage may be consummated. Nowadays, as our laws are becoming less pliable, and venereal diseases must be reported (at least in certain States), he cannot afford to gamble; the absence of the gonococcus must be proven if the patient is to escape quarantine. The ulcer upon the corona glandis may not be indurated, but if the practitioner would make safe his position, he must prove that the treponema is absent from that lesion.

Ofttimes it is desirous that he determine whether or not the male is really sterile; and in certain of these cases the physiological test is not applicable. Thus he must fall back upon the laboratory. With these points in view, and with the advice of men interested in the subject of eugenics, I have described below some methods which may easily be applied by the practitioner in the solution of these problems.

*The Duty of the Sober Physician.*—The subject brings to mind a conversation which I had with a farmer who had been experimenting in raising animals to sell to laboratories. Although he became intensely interested in the subject, he soon found that it was not entirely free from responsibilities.

"Uncle," I asked one day, "in your experience, which do you believe has the most sense, a guinea pig or a hen?"

He thought about the matter for a long time and then drawled disgustedly: "Wall, Doc, I don't think it's a question as to which has the most sense quite so much as to which has the least."

The answer was a good one and may be applied almost every day. The ultra-scientific, pseudo-medical man upon one hand and the sloppy diagnostician upon the other—each a dangerous extreme—make up Medicine's chief disgraces. As the sober physician informs himself in these matters, the former will desert our great science and confine his "researches" to other fields, while the latter will be forced to devote the remaining days of his life to vocations other than medicine.

I have long claimed that the best laboratory methods are the simple ones; and the practitioner has but little sympathy to expect from the future if he fails to make good in these. There is a tendency upon the part of this race to make itself a clean one, and by this means seek its preservation. The medical man who lags behind will have only himself to blame when he falls so far in the rear that it is useless to attempt to regain lost ground.

*Identification of the Gonococcus.*—Although we are able to cultivate the gonococcus upon artificial media, so far as purely diagnostic purposes are concerned it is only necessary that we examine smears of the suspicious material, by aid of the microscope, and attempt to identify this germ. It is not necessary that I go into the various methods of obtaining this material, for such falls into the sphere of bedside or office work. But a few words may make clear one of the best procedures to apply in chronic cases where no morning drop is seen.

The patient's supper consists of foods and drinks which have previously been debarred. Among these I would mention pork. A few drams of gin or a couple of beers may be taken with diagnostic profit, if the scruples of the patient (some very nice people get a dose now and then) do not contraindicate. In the morning, the prostate and seminal vesicles are massaged and an attempt is made to express and smear a droplet of exudate from the external meatus. Then the urine is passed, and the sediment from this is also smeared. The vagina of the female rarely contains gonococci; but smears must be made from the cervical canal and from the urethral meatus.

I am inclined to believe that while cultural methods are never necessary for the identification of the gonococcus in its venereal haunts, they are always imperative elsewhere, as in gonorrheal septicemia, gonorrheal meningitis, etc. However, the venereal diagnosis being the usual one, it is fortunate for the practitioner that smear examinations give sufficient information.

But if we would demonstrate the presence of the gonococcus, we must at least make smears. What is a smear? If a droplet (*not a drop*) of the suspicious material be placed upon a microscopic slide or piece of window glass and spread in such a manner that the thinnest film is formed upon the surface of the glass, such a film is called a smear. By properly treating this smear, the gonococci may be seen through the microscope.

*Smearing and Fixing the Material.*—How is a smear prepared? I can conceive of nothing quite so simple as pus smearing; and yet it is rarely done correctly. Since smears are so necessary in all kinds of bacteriological work, especially in that which may be applied by the physician, I have thought it well to give a description of the method of smearing which has served me best.

A droplet (most men use a drop and therefore fail to see through the smear after it is stained) is placed upon a corner of a clean slide or a piece of window glass. By means of a clean wooden toothpick, this droplet is drawn gently into a short line across the end. Then part of the toothpick is laid into this in such a manner that they coincide. Gently it is swiped over the surface, thus making a thin and almost transparent film. This film is permitted to dry in the air and the toothpick is burned.

SMEARING OF EXUDATES.

1. First step—A droplet is placed in corner of slide.
2. Second step—By means of the end of a clean toothpick this droplet is drawn into a line.
3. Third step—Then the toothpick is laid in this line and drawn across the face of the glass.

Before proceeding further with this technic, let us tarry to review some of the methods usually applied and which account for so many bad results. Some men make the spread by means of the sharp end of the toothpick, thus breaking up the cells and smearing it very unevenly—thick in some portions and thin in others—so that when examined we have no idea as to the relative numbers of gonococci which may be present. Some men scarcely spread the material, and after it is stained it is so opaque that an X-ray would be necessary to see through it. Now and then men place a drop of the pus between the faces of two slides and send to laboratories in this fashion. I fail to understand how such an idea ever found root in the mind of the practitioner. Of course such a preparation is invariably refractory to all examinations.

After the film has *entirely dried in the air,* the practitioner holds it with the thumb upon one edge and the second finger upon the opposite one, with the smear face up. Thus he carries it gently with his hand into the blue flame of his alcohol lamp, slowly through and then out, repeating the process several times, heating the glass so that it is *hot but not hot enough to brown the film.* After a little practice this point will soon be learned. This heating is called fixing and serves to glue the preparation to the glass so that it may be stained. If the practitioner sends his smears to a laboratory for expert opinion, he may omit this heat fixation and wrap the slide, after the film has dried in the air, in a piece of heavy paper. Fixing and staining are done at the laboratory. In sending slides to a laboratory, each should be wrapped separately and should never be pressed together with the films directly in contact.

*Selection of a Staining Method.*—Having fixed the film, the practitioner is now ready to stain it; and after he has finally decided upon which method he prefers to use he will have most of the work completed, for the staining of the gonococcus is a

"pipe." I wish to spend some time upon the matter of the selection of a staining method. The gonococcus may be identified in smears by means of its morphology alone when treated by the simple stains and by means of its staining reactions when treated with the more complex stains; so that this is the point which will guide the physician in the selection of his method.

Morphologically the gonococcus is of a coffee-bean shape, or plano-convex. As a rule, two of the cocci are usually found practically in contact; and to this appearance has been given the name diplococcus. Upon close examination it will be found that their plane surfaces are apposed, and the impression is gained that two spheres have been pushed together in such a manner as to flatten their approximating surfaces, like as two biscuits are pushed together, thus leading to the crude expression, "biscuit-bugs." There is a tendency upon the part of the gonococcus to occur in bunches. In pus, these groups are often found within the protoplasm of the cells, and this tendency alone often identifies the gonococcus. However, groups may be found outside the pus cells; and in chronic gonorrheal urethritis the squamous cells alone may contain them.

Morphologically the gonococcus may be confused with but one other micro-organism, the diplococcus urethræ communis; but the practitioner should have no real difficulty here. This latter germ is larger and longer, never lies within the pus cells and rarely in the epithelial cells; it is always of practically constant shape and size, whereas the size and to some extent the shape of the gonococcus varies, especially in the acute urethritis. With a little practice the physician will not need to resort to the differential staining methods.

The simple methods which I believe to be of most value, are the Loeffler's stain and the neutral red stain. Both of these will be described and discussed below.

We have found that the gonococcus may be differentiated by its staining properties; and while this is a more certain means of identifying the gonococcus by one who becomes expert in methods but not in eye, I hesitate to recommend these methods to the practitioner as routine procedures, inasmuch as the technics are difficult and the sources of error, for this reason, much greater.

I am inclined to believe that two smears should always be made. If the first fails to give conclusive results by the simple methods, the contrast stain may be applied to the other. The chief method of contrast staining is that of Gram, this being used in many other fields of bacteriological work. The technic has been given below.

Fortunately, however, one of our simple methods does very nicely for contrast staining. This is the neutral red method described below and which I am about to recommend very highly for this work.

*Loeffler's Method—Technic, Value and Limitations.*—I have stated in my book that the use of Loeffler's stain is sufficient for practical purposes, and I am not inclined to retract this assertion. Of course, it was meant to hold only so far as diagnosis of urethral smears is concerned. The gonococcus, once met, is easily remembered and frequent introductions are not necessary. His personal appearance is perhaps more characteristic than is his demeanor; and his confusion with the diplococcus urethræ communis rarely justified by any man who has seen both. Given a smear presenting typical gonococci, may we swear upon the witness stand that the person has gonorrhea? Yes, providing we know that the smear was taken from the genitals. In sputum or in meningeal exudate, the case would be different, but no confusion should exist if the source of the smeared material be known.

Loeffler's methylene blue solution may be purchased already prepared from any of the optical or chemical houses. A fluid ounce costs a quarter and will last for years. Enough of this solution to cover the film is dropped upon it and remains there for a couple of minutes. Then the excess stain is washed off under the tap or in a basin of water, the slide paddling gently the water. It is dried *gently* between two ordinary blotters, and this completes the preparation. It is not necessary to add a cover glass. When using oil immersion lens, drop the cedar oil directly upon the film.

The first thing which catches the eye of the practitioner upon looking through the scope is the large number of blue splotches. The larger of these are the nuclei of the cells, and especially of the pus cells; and it will be seen that two or three of these lie within each cell. By looking a bit more closely, the gonococci may be distinguished, likewise stained blue, but much more deeply. The size of the gonococcus, taken roughly, is to that of a nucleus in much the same ratio as a hazel nut is to an orange. These gonococci usually lie very near the nucleus and in the protoplasm of the cell, but sometimes outside the cell. The morphological features have been described above. The oil immersion lens should be used wherever possible.

If the film is not stained, it probably was not sufficiently fixed, and the cells have been washed off the glass. If, upon the other hand, the film was overheated, the cells were destroyed (burned).

*A Simple Stain With Differentiating Powers.*—Several other simple staining methods may be used for identifying the gonococcus, but the same principles are involved and directions may be slightly modified to fit these. Arneill describes a very simple method which I have applied with considerable success. In a dilution of about 1-15,000 it has been found that neutral red shows a selective action for the gonococcus, while the diplococcus urethrae is but feebly stained. Buy the dry powder of this stain (a very little will last a lifetime) and have your druggist make up a one per cent solution in distilled water. Filter this and add a drop of formalin to preserve it. Label it "stock solution." When you are ready to stain the smear, add enough of this stock solution to a tumbler of distilled water to give a sherry wine color (a dessert spoon to an ordinary tumbler will usually give this color). After the solution is mixed, the fixed slide is set upon end in the tumbler in such a manner that the film is bathed in the solution, but does not come into contact with the side of the vessel. After five minutes it is removed from the solution, washed *quickly* by paddling gently some distilled water in another tumbler, dried gently between two ordinary blotters, and examined as above. The gonococci are stained red and the urethral diplococcus, if present, is stained pink. Other germs are not stained.

*Gram's Stain.*—Inasmuch as Gram's method of staining bacteria is not usually made clear to the practitioner in most books upon laboratory work, I shall include a description. The advantages and disadvantages have been discussed above. The application of the method is not nearly so difficult as is generally believed, but the sources of error are considerable unless the physician becomes experienced in the work. As a rule, I would recommend any other method but the Gram's, although I am free to confess that this opinion is not a universal one. Recommendations of this method are made much too strong. In the first place, it is time-consuming; and, in the second place, the practitioner with a little experience *can always recognize the gonococcus by the other methods.*

1. Upon the fixed film, pour some carbol gentian violet solution. (This solution may be obtained in ounce bottles.)

2. After two or three minutes, pour off this stain and add at once enough Gram's iodin solution to cover the film. It is well to add some more fresh iodin solution after a minute. When the coffee grounds color appears, or after the iodin has been on for about ninety seconds, wash in water under tap or in a basin by the method suggested for Loeffler's stain. (Gram's iodin solution may be obtained in ounce bottles.)

3. Shake off the water and add 95 per cent alcohol, leaving this on the film until no more violet color soaks out.

4. Wash again in water.

5. Stain in neutral red as directed above. The gonococci will be stained red and the other germs violet. This method of Gram's staining is reliable and best suited to the needs of the practitioner, inasmuch as no heating is necessary and the complicated aniline solutions are avoided.

*Accurate Diagnosis of Syphilis.*—Fortunately, in this country, lues is not a common disease as compared to gonorrhea. The Wassermann serodiagnosis of syphilis must as a rule be carried out in a laboratory and by a man who does much of the work, not only because he is best fitted to interpret results, but because the occasional Wassermann is very expensive and time-consuming.

The physician is not justified in diagnosticating chancroid unless the treponema pallidum is persistently absent. Much too often does it happen that syphilitic sequelæ follow soft sore. I have taken such great pains in other communications to describe the office identification of the treponema that I do not feel justified in taking the space here. Although not so frequently met as gonorrhea, the disease is an important one, and the physician must be prepared to "clear his skirts" if he fails to report cases of syphilis, believing that such is not the diagnosis.

*Sterility in the Male.*—Ofttimes it is necessary to make a microscopic examination of the semen to determine sterility. Some men who are sterile may possess spermatozoa, but these not be motile. In such a case, the examination must, of course, be made before the semen is allowed to cool. Under these conditions, several examinations are necessary to prove that sterility is absolute, as other spermatozoa loosed a week or so later may be actively motile.

A droplet (not a drop) of the semen is placed upon a clean slide or a piece of window glass and a clean cover glass placed upon this, flattening it gently into a thin film. Stains are not used, but the preparation is examined while wet. It may be wise to leave off the cover glass if there is any question in regard to the motility; but under such conditions the oil immersion lens cannot be used unless we prepare a hanging drop in a concave slide. After the proper focus is obtained, it may be well to contract the iris diaphragm somewhat in order to identify these elements.

